# Lipid Profiles, Glycated Hemoglobin, and Diabetes in People Living at High Altitude in Nepal

**DOI:** 10.3390/ijerph14091041

**Published:** 2017-09-10

**Authors:** Nirmal Aryal, Mark Weatherall, Yadav Kumar Deo Bhatta, Stewart Mann

**Affiliations:** 1 Department of Medicine, University of Otago, Wellington 6021, New Zealand; mark.weatherall@otago.ac.nz (M.W.); stewart@mannz.co.nz (S.M.); 2 Norvic International Hospital, Kathmandu 14126, Nepal; ykdbhatt@yahoo.com

**Keywords:** lipid profile, total cholesterol, triglyceride, high-density lipoprotein-cholesterol, low-density lipoprotein-cholesterol, glycated hemoglobin, diabetes, high altitude, Nepal

## Abstract

This study aimed to describe lipid profiles and the distribution of glycated hemoglobin (HbA1c) in a sample of a high altitude population of Nepal and to explore associations between these metabolic risk variables and altitude. A cross-sectional survey of cardiovascular disease and associated risk factors was conducted among 521 people living at four different altitude levels, all above 2800 m, in the Mustang and Humla districts of Nepal. Urban participants (residents at 2800 m and 3620 m) had higher total cholesterol (TC) and triglyceride (TG) than rural participants. A high ratio of TC to high-density lipoprotein-cholesterol (HDL) (TC/HDL ≥ 5.0) was found in 23.7% (95% CI 19.6, 28.2) and high TG (≥1.7 mmol/L) in 43.3% (95% CI 38.4, 48.3) of participants overall. Mean HbA1c levels were similar at all altitude levels although urban participants had a higher prevalence of diabetes. Overall, 6.9% (95% CI 4.7, 9.8) of participants had diabetes or were on hypoglycaemic treatment. There was no clear association between lipid profiles or HbA1c and altitude in a multivariate analysis adjusted for possible confounding variables. Residential settings and associated lifestyle practices are more strongly associated with lipid profiles and HbA1c than altitude amongst high altitude residents in Nepal.

## 1. Introduction

Dyslipidemia and diabetes mellitus (DM) are key global public health problems. Hypoxic and hypobaric conditions of high altitude (HA) alter the use of energy producing metabolic fuels [1,2] which may secondarily affect lipid and blood glucose concentrations. Hypoxia causes a preference for glucose utilization and decreases uptake of free fatty acids because of oxygen-efficient adaptation [1,3]. Hypoxia also alters hepatic lipid oxidation and can increase the levels of triglyceride (TG) [4] and high-density lipoprotein-cholesterol (HDL) [5].

More than 140 million people in the world permanently live at HA, comprising 2% of the global population [6]. Usual residence at an altitude of 2500 m or above is the conventional demarcation for HA [7] because people generally start to experience adverse symptoms such as shortness of breath, increased heart rate, nausea, dizziness, satiety and fatigue above this altitude. The largest populations at HA are 80 million in the Himalayan mountains of Asia and 35 million in the Andean mountains of South America [6].

In Nepal, almost 2 million people live permanently in mountainous areas, representing 7% of the national population [8]. A large proportion of the population of the hilly districts are also resident at altitudes greater than 2500 m. Earlier estimates are that around 35% of the total Nepalese population permanently live at higher than 2500 m of elevation [6]. Two major ethnic populations living at HA in Nepal are Tibetans and Khas-Aryas. Particularly those from Tibetan origins (such as Sherpa and Thakali) have culturally derived food practices of consuming large amounts of alcohol, salt, meat products and oil [9]. We hypothesized that physiological responses from chronic exposure to the hypoxic environment of HA may result in adverse effects on lipid profiles and diet and lifestyle factors of HA residents of Nepal may increase average blood glucose levels, despite metabolic adaptation. To our knowledge, no previous study has described lipid profiles or the distribution of glycated hemoglobin (HbA1c) and diabetes at particular levels of HA in Nepal.

## 2. Methods

### 2.1. Study Area and Sample

There are 16 districts classified as mountain ecological regions of Nepal, and two of these mountainous districts, Mustang and Humla, were purposively selected for sampling based on past documentation of geography, altitude, population and ethnicity. Random sampling of the districts was not possible due to the issue related with altitude level (some are below 2500 m) and logistical support (availability of electricity and local transport) to conduct the study. However, the selected districts are similar to other mountain districts of the country as they represent both urban and rural settings and include major HA ethnic populations: Tibetans and Khas-Aryas. The selected study areas were Jomsom (2800 m), which is an urban setting, Jharkot (3270 m), a rural setting, Muktinath (3620 m), another urban setting of the Mustang district, and Simikot (2890 m) which is a rural setting of the Humla district. Urban areas within Mustang district and rural areas of Mustang and Humla districts are hugely comparable in terms of their socio-economic status, lifestyle, livelihood, and health practices. The population with Tibetan ancestry is predominant in Mustang district, whereas a Khas-Arya population constitutes the majority in Humla district. Tourism and its natural resources mean that Mustang people are relatively wealthy and the dominant Thakali ethnic group has the highest human development index (HDI) score in Nepal [10]. In contrast, the Humla district has the lowest HDI score [10] and it is the only district of Nepal without any road access. Both districts border Tibetan areas of China and the Tibetan culture is influential in these districts. Life expectancy at birth is 64.1 years in Mustang and 65.0 years in Humla, both lower than the national figure of 68.8 years [10].

Tibetans migrated to high mountains of Nepal about 500 years ago. Tibeto-Burman is their native language and they practice Buddhism. Khas-Aryas migrated to Nepal about 2000 years ago, speak the Nepalese language, and the majority of them are Hindu. Tibetans and Khas-Aryas have distinctive physical appearances, languages, and names.

The inclusion criteria for study participants were age 30 years and above and the ability to speak and understand the Nepalese language. Exclusion criteria were the inability or unwillingness to provide written or verbal consent, inability to speak or hear properly, and pregnancy.

### 2.2. Sampling Process

Sampling was carried out in two stages. In the first stage, three study areas of Mustang and one study area of Humla were selected on the basis of altitude levels, population density and logistical support to undertake the study. In the second stage, a list of households was developed, and a unique number was assigned to each of them. Household numbers were randomly selected with the help of a computer based randomization technique. All eligible family members of the randomly selected households were considered. If none of the household members were eligible or agreed to take part in the study, a household in the close proximity was selected.

The desired sample size within each district was calculated to be around 250 individuals. This study was part of comprehensive cardiovascular risk assessment study and the sample size was based on the precision to estimate the prevalence of hypertension (HT). This sample size gave a margin of error of plus or minus 5% for a proportion based on anticipated prevalence of HT of around 25% after adjusting for the design effect and an expected non-response rate of 10%. We estimated that precision levels would be very similar for all reasonably prevalent variables (including lipid profiles and diabetes).

### 2.3. Data Collection Procedures

We administered the World Health Organization’s STEPS questionnaire version 2.2 for non-communicable disease risk factors to consenting participants. The Nepal Health Research Council (NHRC) had already translated this into the Nepalese language and it has been validated through a pilot study in a community and approved by expert meetings [11]. Administration of the questionnaire and blood pressure measurements were carried out at the house of the selected participants. Practically it was not feasible to undertake blood sample testing for lipid components and HbA1c, and bio-physical measurements in individual houses so a local community hall was used as the measurement facility. Participants were requested to come for these measurements at a time suitable for them from the next day. HbA1c and lipid profiles were measured using Cobas b 101 device (instrument code 06378668190, Roche Diagnostics International Ltd., Rotkreuz, Switzerland). A small amount of capillary whole blood was taken by single finger-prick and processed by the device. A quality control check of this device was performed after every 100 participants, using the manufacturer’s operating guidelines.

Data was collected between June and August (summer) 2014 in Mustang district and March and May (spring) 2015 in Humla district. The ambient temperature was between 25 °C and 31 °C in Mustang and around 15 °C in Humla on most of study days.

This study was approved by the ethical review board of NHRC and the University of Otago, Human Ethics Committee (Nepal Health Research Council–62/2014 (date of approval 15 May 2014), University of Otago Human Ethics Committee (Health)–H14/056 (date of approval 14 April 2014)).

### 2.4. Measures

A person was classified as having diabetes or pre-diabetes if his/her HbA1c was ≥48 mmol/mol or ≥39 to 47 mmol/mol, respectively, according to the guidelines of the American Diabetes Association [12]. The National Cholesterol Education Program (NCEP) and Adult Treatment Panel III (ATP III) guidelines were used to classify lipid profiles [13], according to which a person was classified as having high total cholesterol (TC), high TG, high low-density lipoprotein-cholesterol (LDL), and high non-HDL if this level was ≥5.2 mmol/L, ≥1.7 mmol/L, ≥3.4 mmol/L, and ≥4.1 mmol/L, respectively. HDL was classified as low if this level was <1.3 mmol/L for women and <1.0 mmol/L for men. High TC to HDL cholesterol was defined as the ratio ≥ 5.0.

### 2.5. Statistical Methods

Simple data description is by mean, standard deviation (SD), frequency counts, and proportions expressed as percentages. The Clopper-Pearson method was used to estimate an exact confidence interval for a single proportion. Analysis of covariance (ANCOVA) models were used to estimate the associations between TC to HDL ratio and HbA1c with altitude. We analyzed data available for completed TC to HDL ratio and HbA1c results. Altitude was treated as a continuous variable in the multivariate models. Stata version 12 was used for data analysis.

## 3. Results

A total of 521 HA residents at four different altitude levels participated in this study; 2800 m (*N* = 165), 3270 m (*N* = 61) and 3620 m (*N* = 44) of the Mustang district and 2890 m (*N* = 251) of the Humla district. Due to the instrument failure at 2890 m of the Humla district, lipid profile data was available only from 168/251 (67%) participants and HbA1c data from 171/251 (68%) participants at this level. Those with missing data on lipid profile and HbA1c were significantly more likely to be young and from rural areas, whereas for HbA1c a significantly higher proportion of men had missing data compared to women. More than 70% of participants in Mustang were Tibetans and >70% in Humla were Khas-Aryas. Particular ethnic groups amongst Tibetans were: Thakali, Tibetan Gurung and Lama; and for Khas-Aryas: Brahmin, Chhetri, and Dalit. Participants are described in Table 1.

More than a quarter of the study participants at altitudes of 2800 m, 3270 m and 3620 m were aged 60 years or more. There was evidence of a difference in age at the different altitude levels; Kruskal-Wallis test Chi-square (3 DF) 59.1, *p* < 0.01. Women comprised a greater proportion than men at all levels of altitude and overall 55.7% of study participants were women but there was no evidence this proportion was different by altitude level; Chi-square (3 DF) 2.5, *p* = 0.47. Most of the participants were illiterate or had no formal education and reported being born and living at HA for most of their lives. By self-report about half of the participants at all altitudes stated they were current consumers of alcohol.

Lipid components, HbA1c or diabetes-related variables are presented in Table 2 by altitude levels. Residents in urban settings at 2800 m and 3620 m had higher TC and TG than those in rural settings. Those resident in rural settings at 2890 m had a lower HDL to those in urban settings at 2800 m. Nearly one-quarter of the participants, 23.7% (95% CI 19.6, 28.2), had a high TC to HDL ratio. Based on cut-points, abnormal results for at least two lipid variables were found in 35.3% (95% CI 28, 36.9) of participants. More than half of the participants living in urban settings at 2800 m and 3620 m had high TG and overall in 43.3% (95% CI 38.4, 48.3). Only four (0.8%) participants self-reported being treated with cholesterol lowering drugs. Hazardous drinkers were more likely to have a higher risk of abnormal lipid values and diabetes compared to non-hazardous drinkers (Appendix A).

Mean HbA1c measurements were similar at all altitude levels; however, participants living in urban settings at 2800 m and 3620 m had a higher prevalence of diabetes than those in rural settings. In contrast, pre-diabetes was more prevalent in those living in rural settings at 2890 m and 3270 m than urban settings. Nearly one-third of the participants overall had pre-diabetes: 30.9% (95% CI 26.5, 35.6). Three-quarters (74.5%) of the participants had never had a blood sugar test before and 2.7% were currently on anti-diabetic treatment.

Those living in urban settings compared to rural settings had significantly higher proportions of participants with high TC, Chi square (1 DF) 5.4, *p* = 0.02; high TG, Chi square (1 DF) 13.3, *p* < 0.01; and diabetes, Chi square (1 DF) 9.9, *p* < 0.01 (test for associations not shown in Table 2). The prevalence of pre-diabetes was significantly higher in rural settings compared to urban settings, Chi square (1 DF) 10.5, *p* < 0.01.

**Figure 1:**
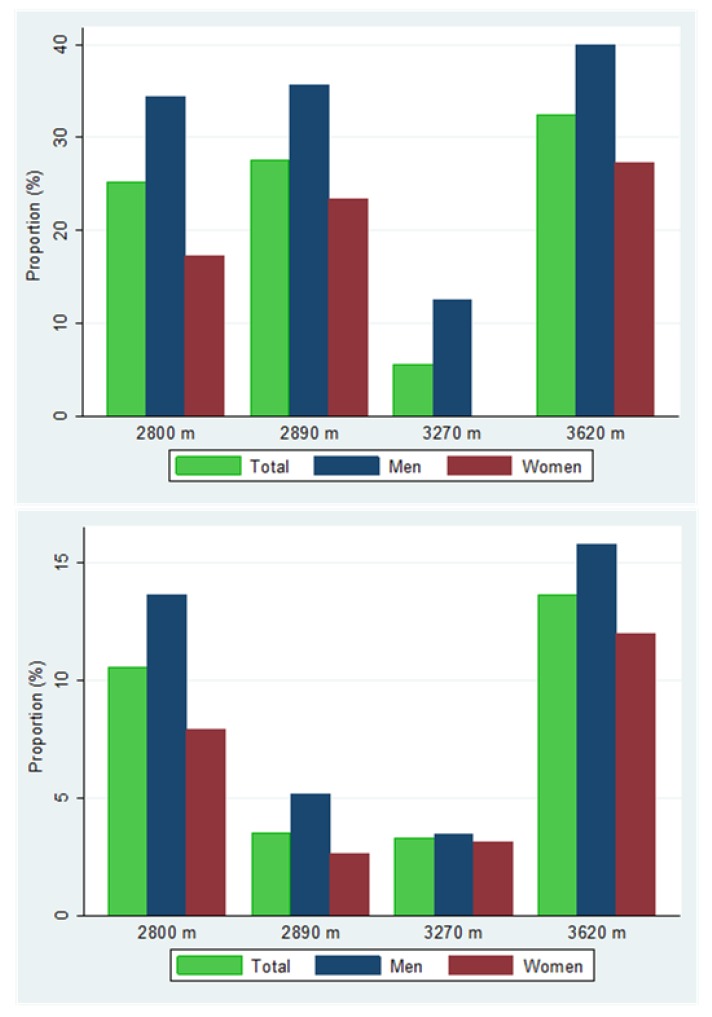
Proportions of high total cholesterol (TC) to high-density lipoprotein-cholesterol (HDL) ratio or on cholesterol treatment (above) and diabetes or on treatment (below) by sex at each altitude level.

A higher proportion of men had a high TC to HDL ratio, or were on treatment, at all altitude levels (Figure 1); however, the difference in proportions compared to women was statistically significant at 2800 m only: proportion difference of 16% (95% CI 2, 30), *p* = 0.03. Men were also more likely to be diabetic, or on treatment, than women at every altitude level (Figure 1), but the differences in proportions between men and women were not statistically significantly different at any altitude level.

In multivariate ANCOVA models adjusting for potential confounding variables, there was no evidence of a relationship between TC to HDL ratio, HbA1c, and altitude. The estimate of the mean TC to HDL ratio (95% CI) per 1000 m higher altitude was −0.4 (−0.9, 0.09), *p* = 0.10 (Table 3). Male sex and higher body mass index (BMI) were associated with higher mean TC to HDL ratio but being a current drinker was associated with lower mean TC to HDL ratio. The relationship between mean HbA1c and altitude was positive but with a wider confidence interval. For every 1000 m elevation of altitude, mean HbA1c increased by 0.1 mmol/mol (95% CI −2.6, 2.9), *p* = 0.92 (Table 4). As might be anticipated, older age and higher BMI were associated with higher mean HbA1c. Sensitivity analysis excluding outlier observations did not change overall conclusions. Separate analyses including hazardous drinking patterns in both models did not change overall conclusions as well; however, the effects of altitude decreased for TC to HDL ratio and increased for HbA1c (Appendix A).

## 4. Discussion

In this study, there was no evidence of statistically significant associations between TC to HDL ratio, or HbA1c, and altitude. The direction of the effect was positive for HbA1c and negative for TC to HDL ratio but with wider confidence intervals. This study, however, indicates a high prevalence of dyslipidaemia and raised blood glucose levels in HA residents of Nepal. For example, one-fourth of the participants had high TC to HDL ratio and more than one-third had high TG. Although the prevalence of diabetes is comparable to the worldwide prevalence [14], nearly one-third of the participants had pre-diabetes, particularly so among those living at rural areas. Similar to findings from the general adult population, men were more likely to have dyslipidemia and diabetes in this study as well.

No clear relationship between lipid components and long-term residence in HA has been identified by previous studies [15,16,17,18]. The most consistent finding is that long-term living at HA is associated with increased TG levels [18,19,20]. The present study also identified a higher prevalence of a high TG level compared to the national prevalence of Nepal for populations aged 30 years or above (33.4%) at all altitude levels except 2890 m [11]. This is likely to be related to high consumption of alcohol and carbohydrates [21,22]. In addition, alteration in hepatic lipid oxidation in hypoxia induced by HA may also increase TG levels [4].

As for the lipid components, evidence for effects on blood glucose by HA living is also inconsistent and varies by population [23,24,25,26]. The reasons for high prevalence may be hypoxia induced polycythemia [23] and changing from traditional lifestyle and dietary habits to other lifestyle habits. Lifestyle factors may overwhelm protective effects induced by physiological phenomena in some populations. The main causes for low prevalence of diabetes may be increased preference for the use of glucose as a metabolic substrate [1,2,3], lower socio-economic status of HA residents [27], higher insulin sensitivity compared to low altitude residents [28], anti-diabetic properties of plants and crops produced in HA [29], and cold ambient temperature affecting glucose metabolism during shivering [30].

In the present study, the prevalence of diabetes was higher than past estimates for the Nepalese population, particularly those in rural areas [31,32]. Although differences in definitions and the inclusion of only participants aged 30 years or over may partly explain the difference when compared with other estimates, the increasing risk of diabetes in HA populations in the present study was unsurprisingly accompanied by a higher rate of pre-diabetes. The prevalence of pre-diabetes was nearly three-fold higher than the national estimate of 10.3% [32] and particularly in rural settings (2890 m and 3270 m). While moderate drinking has been found to have a protective effects on blood glucose levels, evidence is strong on the relationship between heavy drinking and the risk of diabetes [33,34]. A significant proportion of rural dwellers in the present study were hazardous drinkers (men drinking ≥ 21 standard drinking unit (SDU) and women drinking ≥ 14 SDU of alcohol per week); for example, 56.4% of participants at 3270 m self-reported being hazardous drinkers. This may partly account for the very high prevalence of pre-diabetes in rural dwellers in the present study.

Urban dwellers (2800 m and 3620 m) have a higher risk of dyslipidemia and diabetes compared with rural dwellers (2890 m and 3270 m), even in similar ethnic populations. This may be due to higher economic status and obesity in the urban participants. This suggests that lifestyle practices are more important than altitude and ethnicity in determining the metabolic risk of HA residents of Nepal. Previous studies in Tibet also indicate desirable patterns of plasma lipid components in rural HA residents [16] compared to the urban HA population [15]. The findings of a higher prevalence of dyslipidemia, diabetes, and pre-diabetes in men may be attributed to the cultural differences in dietary and lifestyle habits. For example, in Tibetan culture, men were more likely to participate in social gatherings which involve consumption of alcohol and high fat laden butter tea consumed over several hours.

This is the first study to report the prevalence of abnormal lipid components and diabetes in Nepalese people living at different levels of HA. Just one previous study has reported TC levels and diabetes in mountainous regions but without specifying particular altitudes [35].

Limitations of this study include possible selection bias due to the purposive selection of study areas based on altitude levels, population density and logistical convenience. Lipid profiles and HbA1c were measured using the same instrument and technical problems at 2890 m may have reduced the precision of estimates for this group because of the lower sample size. This survey used non-fasting blood samples for determination of a lipid profile. Fasting is not routinely recommended for lipid profile tests. However, evidence shows a minor increase in TG levels after habitual meals, although clinically not significant [36]. Thus, the present study might have over-reported TG levels in some participants with lipid profile measurements after meals. A 'healthy individual effect' may bias our findings because less healthy individuals who could potentially have been part of the sampling frame may have moved away to low altitude areas for better quality health care. On the other hand, migration of young and healthy populations to city areas of the country or emigration to other countries for jobs or education is common in study areas. Thus, this study might have missed potentially eligible healthy participants.

## 5. Conclusions

In this study, there was no clear association between lipid profiles or HbA1c and altitude in a multivariate analysis adjusted for possible confounding variables. Residential settings and associated lifestyle practices are more strongly associated with lipid profiles and HbA1c than altitude amongst HA residents in Nepal. The HA residents of this study are at increased risk of dyslipidemia (particularly high TC to HDL ratio and high TG) and pre-diabetes. This study has also corroborated previous reports of high TG levels in HA populations. This is the first study to report the prevalence of abnormal lipid components and diabetes in HA areas of Nepal at specified levels of altitude. More evidence on the risk of dyslipidemia, diabetes and its risk factors among different ethnic HA populations in Nepal and at different levels of altitude may lead to appropriate health interventions. Clinicians should be aware of high TG levels and particularly a high rate of pre-diabetes in HA residents of Nepal.

## Figures and Tables

**Table 1:** Description of study participants by altitude level.

District		Mustang		Humla	
Altitude	2800 m	3270 m	3620 m	2890 m	
	(Urban)	(Rural)	(Urban)	(Rural)	
	(*N* = 165)	(*N* = 61)	(*N* = 44)	(*N* = 251)	
**Continuous variables**	**Mean (SD)**				***p* Value**
Age	50.3 (13.7)	55.4 (13.2)	48.3 (12.5)	42.9 (11.0)	<0.001
Average walk time/day (minute)	53.0 (44.8)	95.6 (79.9)	73.2 (53.0)	89.4 (89.6)	<0.001
Average vegetable and fruit consumption/day (portion)	3.7 (1.9)	2.7 (1.2)	3.2 (1.2)	4.5 (1.8)	<0.001
**Categorical variables**					
	*N*/165 * (%)	*N*/61 * (%)	*N*/44 * (%)	*N*/251 * (%)	
Sex					0.47
Male	80 (48.5)	29 (47.5)	19 (43.2)	103 (41.0)	
Female	85 (51.5)	32 (52.5)	25 (56.8)	148 (59.0)	
Education					<0.001
No formal education/illiterate	67 (40.6)	41 (67.2)	29 (65.9)	168 (66.9)	
Less than primary	45 (27.3)	19 (31.1)	6 (13.6)	18 (7.2)	
Primary level completed	25 (15.1)	0 (0)	4 (9.1)	20 (8.0)	
Secondary level completed	28 (17.0)	1 (1.6)	5 (11.4)	45 (17.9)	
Occupation					<0.001
Govt. or nov-govt. employee	25 (15.1)	1 (1.6)	7 (15.9)	39 (15.5)	
Self-employed	86 (52.1)	6 (9.8)	14 (31.8)	21 (8.4)	
Agriculture/daily waged labour	41 (24.8)	39 (63.9)	21 (47.7)	135 (53.8)	
Unemployed/retired/homemaker	13 (7.9)	15 (24.6)	2 (4.5)	56 (22.3)	
Ethnicity					<0.001
Tibetan	132(80.0)	58 (95.1)	37 (84.1)	82 (32.7)	
Khas-Arya	33 (20.0)	3 (4.9)	7 (15.9)	169 (67.3)	
High altitude residence					<0.001
for lifetime	148 (89.7)	59 (96.7)	38 (86.4)	246 (98.0)	
>10 years	17 (10.3)	2 (3.3)	6 (13.6)	2 (0.8)	
5 to 10 years	0 (0)	0 (0)	0 (0)	3 (1.2)	
Current smoker	17 (10.3)	2 (3.3)	4 (9.1)	97 (38.6)	<0.001
Current drinker	84 (50.9)	39 (63.9)	20 (45.4)	123 (49.0)	0.17
Hypertension or medication	76 (46.1)	25 (40.9)	24 (54.5)	73 (29.1)	<0.001
Overweight or obesity	81/143 (56.6)	16/60 (26.7)	21 (47.7)	52/251 (20.7)	<0.001

* Except where indicated; hypertension, ≥140/90 mmHg; overweight, body mass index ≥ 25 kg/m^2^; obesity, body mass index ≥ 30 kg/m^2^; high TC/HDL ratio, ≥5.0; diabetes, glycated hemoglobin (HbA1c) ≥ 48 mmol/mol. *p* values for continuous variables were calculated by analysis of variance (ANOVA) or Kruskal-Wallis test and by Chi-square test for categorical variables.

**Table 2:** Lipid profiles, HbA1c and diabetes related characteristics of the participants at each altitude level.

District	Mustang			Humla
Altitude	2800 m	3270 m	3620 m	2890 m
	(Urban)	(Rural)	(Urban)	(Rural)
**Variables**	**Mean ± SD (*N*)**			
TC (mmol/L)	4.5 ± 0.9 (142)	4.2 ± 0.8 (56)	4.5 ± 1.1 (38)	4.1 ± 1.1 (168)
TG (mmol/L)	1.9 ± 0.9 (140)	1.5 ± 0.9 (56)	2.4 ± 1.5 (38)	1.7 ± 0.9 (168)
HDL (women) (mmol/L)	1.2 ± 0.5 (75)	1.4 ± 0.3 (30)	1.3 ± 0.3 (22)	1.0 ± 0.3 (111)
HDL (men) (mmol/L)	1.1 ± 0.5 (64)	1.3 ± 0.3 (24)	1.1 ± 0.4 (15)	1.1 ± 0.4 (56)
LDL(mmol/L)	2.4 ± 0.7 (135)	2.2 ± 0.7 (53)	2.2 ± 0.9 (34)	2.3 ± 0.7 (164)
Non-HDL (mmol/L)	3.3 ± 0.8 (139)	2.8 ± 0.8 (54)	3.3 ± 1.2 (37)	3.1 ± 0.9 (167)
HbA1c (mmol/mol)	38.7 ± 10.6(141)	38.0 ± 5.4 (61)	39.2 ± 8.8 (44)	38.1 ± 4.9 (171)
	**% (*N*)**			
High TC	23.9 (142)	12.5 (56)	15.8 (38)	13.7 (168)
High TG	52.1 (142)	30.4 (56)	57.9 (38)	36.9 (168)
Low HDL	64.1 (142)	39.3 (56)	55.3 (38)	71.4 (168)
High non-HDL	16.4 (140)	7.3 (55)	18.9 (37)	13.8 (167)
High TC to HDL ratio	24.5 (139)	5.6 (54)	29.7 (37)	27.5 (167)
Diabetes or medication	10.6 (142)	3.3 (61)	13.6 (44)	3.5 (172)
Diabetes	9.2 (141)	3.3 (61)	11.4 (44)	2.9 (171)
Pre-diabetes	22.1 (141)	39.3 (61)	25.0 (44)	36.8 (171)
Diabetes newly diagnosed	3.5 (141)	3.3 (61)	6.8 (44)	0.6 (171)

TC: total cholesterol, TG: triglyceride, HDL: high-density lipoprotein-cholesterol, LDL: low-density lipoprotein cholesterol, HbA1c: glycated hemoglobin, high TC: ≥5.2 mmol/L, high TG: ≥1.7 mmol/L, high LDL: ≥3.4 mmol/L, low HDL: <1.3 mmol/L for women and <1.0 mmol/L for men, high non-HDL: ≥4.1 mmol/L, Impaired glucose tolerance: HbA1c ≥ 39 to 47 mmol/mol, Diabetes: HbA1c ≥ 48 mmol/mol.

**Table 3:** Estimates of the mean TC to HDL ratio for altitude in a multivariate analysis of covariance (ANCOVA) and associations for confounding variables.

Variable and Comparison	Estimate (95% CI)	*p* Value
Altitude (per 1000 m higher)	−0.4 (−0.9, 0.09)	0.10
Age (per decade older)	0.04 (−0.06, 0.1)	0.42
Sex (male compared to female)	0.5 (0.3, 0.8)	<0.01
Residential setting (urban compared to rural)	−0.2 (−0.5, 0.09)	0.19
Walk time (per 30 minute higher)	0.03 (−0.02, 0.08)	0.23
Alcohol intake in the past 30 days (yes compared to no)	−0.4 (−0.7, −0.1)	<0.01
Oil consumption per day (per millilitre higher)	−0.006 (−0.01, 0.002)	0.16
Body mass index (per kg/m^2^ higher)	0.1 (0.07, 0.1)	<0.01

**Table 4:** Estimates of the mean glycated hemoglobin (HbA1c) for altitude in a multivariate ANCOVA and associations for confounding variables.

Variable and Comparison	Estimate (95% CI)	*p* Value
Altitude (per 1000 m higher)	0.1 (−2.6, 2.9)	0.92
Age (per decade older)	1.6 (1.09, 2.2)	<0.01
Sex (male compared to female)	1.3 (−0.2, 2.9)	0.09
Residential setting (urban compared to rural)	−0.8 (−2.4. 0.8)	0.33
Walk time (per 30 minute higher)	−0.04 (−0.3, 0.3)	0.77
Alcohol intake in the past 30 days (yes compared to no)	−1.2 (−2.7, 0.3)	0.11
Body mass index (per kg/m^2^ higher)	0.2 (0.05, 0.4)	0.01

## Appendix A

**Table A1:** Lipid profiles, HbA1c and diabetes related characteristics of the participants by drinking pattern.

Drinking Pattern	Hazardous Drinkers	Non-Hazardous Drinkers
Variables	Mean ± SD (*N*)	
TC (mmol/L)	186.7 ± 36.8 (64)	168.1 ± 34.9 (145)
TG (mmol/L)	183.6 ± 99.1 (63)	157.7 ± 81.2 (145)
HDL (women) (mmol/L)	55.5 ± 10.8 (6)	47.3 ± 11.7 (87)
HDL (men) (mmol/L)	51.5 ± 17.2 (52)	42.4 ± 13.1 (58)
LDL(mmol/L)	96.1 ± 36.6 (56)	90.7 ± 28.5 (143)
Non-HDL (mmol/L)	131.2 ± 40.9 (58)	121.9 ± 34.9 (145)
HbA1c (mmol/mol)	37.9 ± 8.2 (66)	38.2 ± 7.6 (147)
	**% (*N*)**	
High TC	35.9 (64)	15.9 (145)
High TG	56.2 (64)	44.1 (145)
Low HDL	35.9 (64)	54.5 (145)
High non-HDL	31.7 (60)	11.0 (145)
High TC to HDL ratio	22.4 (58)	21.4 (145)
Diabetes or medication	7.6 (66)	5.4 (147)
Diabetes	7.6 (66)	4.1 (147)
Pre-diabetes	24.2 (66)	36.7 (14.7)

High TC: ≥5.2 mmol/L, high TG: ≥1.7 mmol/L, high LDL: ≥3.4 mmol/L, low HDL: <1.3 mmol/L for women and <1.0 mmol/L for men, high non-HDL: ≥4.1 mmol/L, Impaired glucose tolerance: HbA1c ≥ 39 to 47 mmol/mol, Diabetes: HbA1c ≥ 48 mmol/mol, Hazardous drinkers: those who weekly consume ≥21 (men) and ≥14 (women) standard drink units of alcohol.

**Table A2:** Estimates of the mean total cholesterol to high-density lipoprotein cholesterol (TC to HDL) ratio for altitude in a multivariate ANCOVA and associations for confounding variables.

Variable and Comparison	Estimate (95% CI)	*p* Value
Altitude (per 1000 m higher)	−0.006 (−0.8, 0.8)	0.99
Age (per decade older)	0.03 (−0.1, 0.1)	0.66
Sex (male compared to female)	0.5 (0.1, 0.9)	<0.01
Residential setting (urban compared to rural)	0.1 (−0.3, 0.5)	0.56
Walk time (per 30 minute higher)	0.009 (−0.05, 0.07)	0.75
Hazardous drinker (yes compared to no)	−0.2 (−0.6, 0.3)	0.43
Oil consumption per day (per milli litre higher)	0.003 (−0.02, 0.01)	0.65
Body mass index (per kg/m^2^ higher)	0.1 (0.08, 0.2)	<0.01

Hazardous drinker: those who weekly consume ≥21 (men) and ≥14 (women) standard drink units of alcohol.

**Table A3:** Estimates of the mean glycated hemoglobin (HbA1c) for altitude in a multivariate ANCOVA and associations for confounding variables.

Variable and Comparison	Estimate (95% CI)	*p* Value
Altitude (per 1000 m higher)	2.0 (−2.1, 6.1)	0.33
Age (per decade older)	1.2 (0.4, 2.0)	<0.01
Sex (male compared to female)	0.7 (−1.7, 3.1)	0.56
Residential setting (Urban compared to rural)	−0.2 (−2.6, 2.1)	0.85
Walk time (per 30 minute higher)	0.007 (−0.4, 0.4)	0.97
Hazardous alcohol intake in the past 30 days (yes compared to no)	−0.9 (−3.5, 1.6)	0.48
Body mass index (per kg/m^2^ higher)	0.2 (−0.07, 0.5)	0.14

Hazardous drinker: those who weekly consume ≥21 (men) and ≥14 (women) standard drink units of alcohol.
